# l-Acetylcarnitine: A Mechanistically Distinctive and Potentially Rapid-Acting Antidepressant Drug

**DOI:** 10.3390/ijms19010011

**Published:** 2017-12-21

**Authors:** Santina Chiechio, Pier Luigi Canonico, Mariagrazia Grilli

**Affiliations:** 1 Department of Drug Sciences, Section of Pharmacology and Toxicology, University of Catania, 95125 Catania, Italy; chiechio@unict.it; 2 Department of Pharmaceutical Sciences, University of Piemonte Orientale, 28100 Novara, Italy; pierluigi.canonico@uniupo.it; 3 Laboratory of Neuroplasticity, Department of Pharmaceutical Sciences, University of Piemonte Orientale, 28100 Novara, Italy

**Keywords:** l-acetylcarnitine, mood disorders, antidepressant, chronic pain, acetylation, epigenetics, adult neurogenesis, NF-κB, mGlu2

## Abstract

Current therapy of mood disorders has several limitations. Although a high number of drugs are clinically available, as of today, nearly two-thirds of individuals do not achieve full symptomatic remission after treatment with conventional antidepressants. Moreover, several weeks of drug treatment are usually required to obtain clinical effects, a limitation that has considerable clinical implications, ranging from high suicide risk to reduced compliance. The characteristic lag time in classical antidepressant effectiveness has given great impulse to the search for novel therapeutics with more rapid effects. l-acetylcarnitine (LAC), a small molecule of growing interest for its pharmacological properties, is currently marketed for treatment of neuropathic pain. Recent preclinical and clinical data suggested that LAC may exert antidepressant effects with a more rapid onset than conventional drugs. Herein, we review data supporting LAC antidepressant activity and its distinctive mechanisms of action compared with monoaminergic antidepressants. Furthermore, we discuss the unique pharmacological properties of LAC that allow us to look at this molecule as representative of next generation antidepressants with a safe profile.

## 1. Introduction

l-Acetylcarnitine (LAC) is a small molecule of growing interest for its biological and pharmacological properties. Originally, LAC primary function was attributed to its role in the transport of short- and medium-chain acyl groups outside mitochondria, a step required for utilization of fatty acids and glucose [1]. Besides its role in energy metabolism, during the last two decades, a number of studies have shown that LAC actions range from antioxidant, neuromodulatory, and neuroprotective effects to modulation of gene expression [2,3,4,5,6,7,8]. Because of the multiplicity of actions and the excellent safety and tolerability profile, LAC efficacy has been investigated in a number of clinical conditions and neurological disorders (Table 1). The drug is currently marketed for the treatment of neuropathic pain. Recent preclinical and clinical data support LAC antidepressant effects and suggest novel underlying cellular and molecular effects, including epigenetic mechanisms. Herein, we discuss the unique pharmacological properties of LAC that allow us to consider this molecule as a novel antidepressant molecule.

## 2. Role of Endogenous LAC in Energy Metabolism

**Figure 1:**
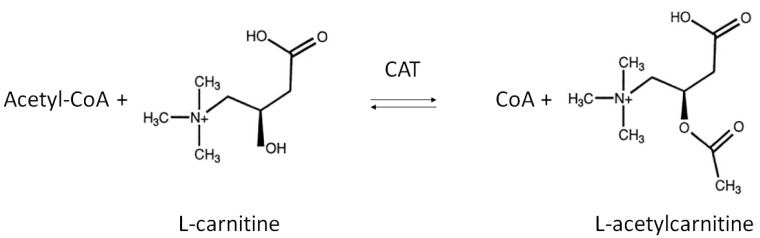
Biosynthesis of l-Acetylcarnitine. The acyl moiety is transferred from acetyl-CoA to the hydroxyl group of carnitine by the enzyme carnitine acetyltransferase (CAT).

LAC is an endogenous compound widely distributed in many tissues, including brain. Chemically, LAC is the acetylated derivative of the amino acid l-carnitine whose function is generally correlated with regulation of energy metabolism within mitochondria [1]. Its de novo synthesis is catalyzed by the enzyme carnitine acetyltransferase (CAT), mainly located on the inner mitochondrial membrane as well as in endoplasmic reticulum and peroxisome [26,27]. CAT promotes the transfer of an acetyl group from acetyl-Coenzyme A (acetyl-CoA) to carnitine, thereby producing LAC and free CoA (Figure 1) [28,29].

After being synthetized, LAC is transported outside mitochondria into the cytosol by the enzyme carnitine/acetylcarnitine translocase (CACT) [30,31,32]. This is a crucial metabolic reaction for β-oxidation of fatty acids whereby LAC facilitates the transport of acetyl-CoA across mitochondrial membranes [1]. A nuclear carnitine acetyltransferase can also convey LAC from cytosol to the nucleus, increasing the local pool of available acetyl groups [33]. It has been estimated that neurons from adult brain contain a relatively high level of LAC, with highest content in hypothalamus [34,35].

## 3. Pleiotropic Effects and Mechanisms for Exogenously Administered LAC

When exogenously administered, LAC is easily absorbed. Due to its amphiphilic structure, LAC is mobile throughout the plasma membranes and can rapidly cross blood-brain barrier [36,37]. Indeed, LAC can be transported by the high-affinity sodium-dependent organic cation/transporter (OCTN2), which is functionally expressed in cells forming the blood-brain barrier [38].

A wide range of mechanisms have been proposed to explain the multiplicity of LAC activities within nervous tissues. In particular, it has been demonstrated that LAC modulates the activity of nerve growth factor (NGF) and enhances the expression of NGF receptors in striatum/hippocampus during development and in aged rats [39,40,41]. Moreover, LAC modulates different neurotransmitter systems, including the gabaergic, dopaminergic, and cholinergic system [42,43,44,45,46], the latter by increasing acetyl-CoA content and choline acetyltransferase (ChAT) activity [47,48,49].

Recently, evidence that LAC serves as donor of acetyl groups further supported additional functions that go beyond its classical role in energy metabolism. In particular, its ability to contribute to acetylation of -OH or -NH2 functional groups on amino acids and N-terminal groups in proteins [2,7,8,50,51] has opened up new avenues to explain mechanisms underlying important LAC activities. Indeed, LAC has been suggested to modulate gene expression by an epigenetic mechanism exerted via acetylation of histone proteins and transcription factors [7,8,50,51,52,53]. Epigenetic mechanisms are physiologically achieved by activity of two classes of enzymes—histone acetyltransferases (HATs) and histone deacetylases (HDACs)—that transfer and remove acetyl groups in histones and transcription factors, respectively [54]. HATs and HDACs were originally identified as chromatin modifying enzymes by operating posttranslational modifications of histones [55]. Later, it was demonstrated that HATs and HDACs do not exclusively target histone proteins but also modulate the activity of a number of transcription factors. Several HAT and HDAC inhibitors are currently under investigation for epigenetic modulation of gene expression. LAC itself, similarly to HDAC inhibitors, regulates epigenetic mechanisms by increasing the acetylation level of histones and transcription factors such as NF-κB [8,51,53,56]. Studies from our groups indicated that LAC regulates the activity of NF-κB signaling pathways by increasing acetylation of the p65/RelA subunit at lysine 310, an event that may enhance transcriptional activity of the protein [7,8]. This effect has been correlated with the ability of LAC to induce expression of metabotropic glutamate receptor type-2 (mGlu2), a potential underlying mechanism for a wide spectrum of pharmacological activities ranging from analgesic, proneurogenic, and antidepressant effects of LAC [7,8,50,51,56,57].

## 4. LAC as a Novel Antidepressant Drug with Unique Properties

In humans, and particularly in the elderly, the beneficial effects of LAC has been reported in mood disorders, including major depressive disorder and dysthymia [58,59,60]. More recently, LAC was investigated in a multicentric, double-blind, randomized clinical trials (RCT) in a population of elderly patients with dysthymic disorder [16]. The drug was evaluated in comparison with fluoxetine for an observation period of seven weeks. LAC and fluoxetine resulted equivalent in their antidepressant efficacy. Of interest, a difference in latency time of clinical response was observed between the two drugs, namely one and two weeks for LAC and fluoxetine, respectively, potentially suggesting a more rapid effect elicited by LAC in humans [16]. Although the rapidity in the onset of LAC therapeutic effects needs to be confirmed in studies with larger sample sizes and in mood disorders other than dysthymia, it is quite interesting in view of the fact that rapid effects have also been observed in preclinical models of depressive-like behavior [51]. 

A recent meta-analysis investigated the effects of LAC on depressive symptoms across published RCT [61]. Again, LAC administration demonstrated efficacy when compared to placebo. Moreover, LAC efficacy was comparable to classical antidepressant agents, but with significantly fewer side effects [61]. These findings are in agreement with another meta-analysis including 34 studies and 4769 patients with persistent depressive disorders. In that analysis, LAC treatment showed lower rates of adverse events and discontinuation than any other drug comparator [62]. In addition, a meta-analysis confirmed that LAC was more effective in older than in younger patients [61]. At present, the reason for a better drug response in elderly people is not clear. Although additional research efforts are required to confirm these findings, the results suggest that LAC may represent a potential alternative to classical antidepressants. Moreover, these clinical observations suggest that LAC may potentially have a mechanism of action that is distinct from conventional antidepressants. Surprisingly, despite these interesting clinical findings, in the past, very few studies have attempted to investigate underlying mechanism(s) of LAC antidepressant effects.

One interesting form of neuroplasticity is adult neurogenesis, the process of generation of new neurons in adulthood. In the dentate gyrus of hippocampus adult neurogenesis occurs in humans across their entire life span [63]. Although reduction of adult neurogenesis per se does not result in depressive-like behavior, it has been proposed that adult hippocampal neurogenesis (ahNG) may be required for some behavioral effects of antidepressants in rodent models and potentially contribute to the antidepressant activity of these drugs in the clinical setting [64,65]. Antidepressant drugs indeed increase hippocampal neurogenesis in rodents [66,67,68], and an increased number of hippocampal neural progenitor cells (NPC) and granule neurons are reported in postmortem brain of depressed patients undergoing antidepressant therapy [69]. In addition, several experimental studies demonstrate that antidepressants can counteract the inhibitory effect of stress on ahNG in rodent models of depressive-like disorder [70,71].

Among regulators of ahNG the NF-κB family of transcription factors has been receiving attention from our and other laboratories [72,73,74,75,76,77,78,79,80,81,82]. In particular, we demonstrated the involvement of NF-κB signaling pathways in the modulation of adult hippocampal neurogenesis in vivo [72] and in the effects of several drugs that are endowed with proneurogenic and antidepressant activity both in vivo and in vitro [78,79,82]. A few years ago, we showed that LAC is a very potent proneurogenic molecule whose in vitro effects on neuronal differentiation of adult hippocampal neural progenitors (ahNPC) are independent of its neuroprotective activity [8]. The in vitro proneurogenic effects of LAC appear to be mediated by activation of the NF-κB pathway and subsequent NF-κB -mediated upregulation of metabotropic glutamate receptor 2 (mGlu2) expression. Indeed, (i) LAC treatment of ahNPC resulted in acetylation of p65 at lys(310) and in mGlu2 protein upregulation, and (ii) LAC-induced mGlu2 expression could be abolished by interfering with NF-κB p65 nuclear translocation [8]. These results prompted us to evaluate LAC effects in vivo in adult mice. We showed that chronic LAC administration blocks depressive-like behavior caused by unpredictable chronic mild stress (UCMS) [8], a preclinical model with face validity and predictivity for human major depression. The utilized dose (100 mg/kg, i.p.) was chosen for its clinical relevance since it corresponds to the lowest recommended dose in humans (0.5 g/day) [83]. Furthermore, that dose regimen effectively increased plasma levels of LAC in chronically treated mice [84]. LAC-mediated behavioral effects correlated with upregulated expression of mGlu2 (and not mGlu3) receptor in hippocampi of stressed mice [8]. Moreover, we demonstrated that chronic LAC treatment significantly increased ahNG [8]. Unlike mGlu2 effects, chronic LAC treatment correlated with increased hippocampal neurogenesis in both stressed and unstressed mice [8]. In this respect, LAC is similar to classical antidepressants that also promote neurogenesis in naive mice when chronically administered [65,66].

In the same year of our findings, a relevant paper confirmed and further extended these observations by demonstrating LAC-mediated antidepressant effects not only following chronic stress, but also in a genetic model of depression, namely Flinders Sensitive Line (FSL) rats [51]. Also in this preclinical model, LAC increased NF-κB p65 acetylation, thereby enhancing *mGlu2* receptor gene expression not only in hippocampus but also in prefrontal cortex [51]. LAC antidepressant effects were long lasting, being still present two weeks after drug withdrawal. On the contrary, in parallel studies, the effects of a classical tricyclic antidepressant like chlorimipramine disappeared after drug withdrawal. Even more strikingly, LAC exhibited antidepressant activity within 2–3 days following administration, compared with 14 days required by chlorimipramine [51]. Of interest, we also observed a remarkably rapid increase in the number of newly generated neurons in hippocampi of LAC-treated mice [8]. In line with Cuccurazzu’s findings, Nasca and colleagues proposed that LAC promotes rapid antidepressant responses, at least in part, via epigenetic mechanisms that may involve acetylation of histone proteins and NF-κB p65, activating in turn *mGlu2* receptor and BDNF gene expression. Of note, a significant reduction of LAC in hippocampi and prefrontal cortex of FSL rats compared to Flinders Resistant Line rats was recently reported, although it was not clear whether LAC mediated antidepressant effects were linked to a correction of its deficiency in those brain regions [85].

Several critical aspects need to be highlighted and further discussed. First of all, LAC is a non-specific acetylating agent and it is unlikely that its antidepressant and proneurogenic actions are solely due to acetylation of nuclear proteins such as p65 and histones. It is possible that the drug acetylates other, even non-nuclear, target proteins. Further studies should be devoted to the identification of those proteins, which may potentially suggest new pharmacological targets.

It should also be underlined that the idea that NF-κB and p65-mediated transcriptional activation per se results in antidepressant or proneurogenic effects is challenged by opposite literature data. Chronic stress augments NF-κB-dependent transcription in the hippocampus [73] and in Nucleus Accumbens (NAc) [86]. In their elegant study, Koo and colleagues demonstrated a critical role for NF-κB signaling in the cellular and behavioral effects of stress via proinflammatory cytokines [73]. Stress was shown to activate NF-κB signaling and decrease neural stem cell proliferation in the adult hippocampus. Moreover depressive-like behavior induced by exposure to chronic stress appeared to be mediated by NF-κB signaling; i.c.v. administration of NF-κB signaling inhibitors resulted in antidepressant activity and prevented the negative effects of stress on hippocampal neurogenesis [73]. Similarly, genetic deletion of another member of the NF-κB family, the p50 subunit, a condition that is associated with increased p65-mediated transcription, correlates with significantly decreased hippocampal neurogenesis in adult mice [72]. On the other hand, we demonstrated that NF-κB -mediated transcription is involved in both proneurogenic and antidepressant effects of other clinically relevant drugs, such as α2δ1 ligands pregabalin and gabapentin [79].

The fact that both induction and prevention of depressive-like behavior and ahNG may rely on activation of NF-κB signaling pathway is likely to reflect the complexity within that regulatory system. The NF-κB protein family is composed of several members, in addition to p65, which can combine to form dimers with different subunit composition which can be differentially activated and exert distinct, even opposite, functions through activation of specific sets of gene targets [87,88,89,90,91]. The p65 as well as other NF-κB subunits can undergo different posttranslational modifications in addition to acetylation, including ubiquitination, phosphorylation, sumoylation, nitrosylation, and methylation [92]. It is possible that specific combination of posttranslational changes within p65 may ultimately dictate distinct NF-κB mediated transcriptional programs associated with induction of depressive-like behavior or with antidepressant effects. In the future, it may be important to identify the full set of NF-κB gene targets activated in the hippocampus and prefrontal cortex in response to stress/LAC treatment, whose products may potentially represent novel biomarkers or targets in mood disorders.

Last but not least, we do not want to infer that NF-κB p65 acetylation and increased mGlu2 gene transcription represent the only mechanisms underlying LAC pharmacological effects. Recent literature data strongly support this idea. In the UCMS paradigm, LAC-mediated reversal of depressive-like behavior was shown to activate a PI3K/AKT/BDNF/VGF signaling pathway [93]. Using endogenously depressed FSL rats, it has been recently reported that oral administration of LAC results in antidepressant-like effects along with improved energy metabolism in the ventral dentate gyrus (vDG) [85]. A detailed transcriptome analysis of vDG identified several metabolic regulatory genes as potential key markers of LAC antidepressant responsiveness and, interestingly, also of predisposition to depressive-like behaviour. More specifically, mineralcorticoid receptor (MR) and leptin receptor (Lepr) transcripts appeared upregulated, whereas mGlu2 and NPY transcripts were downregulated in vDG of FSL rats. Such changes were rapidly corrected by LAC treatment in drug responsive animals [85]. LAC also corrected hyperinsulinemia and hyperglycemia in FSL rats [85].

Other literature data contribute to the concept of pleiotropicity of action for LAC antidepressant effects. It has been proposed that LAC may act as an antidepressant via increased levels of the glia-derived growth factor artemin [94]. Moreover, the drug can increase hippocampal levels of noradrenaline and serotonin, well established positive modulators of adult neurogenesis [80,82,95]. The possibility that the effects of LAC on synaptic energy state may contribute to its antidepressant effects has also been proposed [96]. Finally, regions other than hippocampus and prefrontal cortex may represent the target of LAC pharmacological activity. For example, medial amygdala stellate neurons have been suggested as a novel component in stress response, and LAC has been shown to promote structural plasticity in these cells [97].

## 5. Therapeutic Implications of LAC Unique Pharmacological Profile

Mood disorders are highly prevalent and disabling conditions. Despite a large number of clinically available drugs, as of today, nearly two-thirds of individuals do not achieve full symptomatic remission after treatment with conventional antidepressants [98]. In addition, in treatment responders several weeks (2–6 w) of drug treatment are required to obtain clinical effects, a lag time which has considerable implications ranging from high suicide risk to reduced compliance [98]. Even when remission is achieved, the majority of individuals also suffer residual symptoms, including chronic pain and cognitive impairment, which further contribute to the disease burden. At present, one clinical study in dysthymic patients has proposed that LAC may be more rapid in its therapeutic effects than the selective serotonin reuptake inhibitor fluoxetine. This finding is preliminary, but it is quite interesting that in animal models, drug effects were also more rapid when compared to the tricyclic antidepressant chlorimipramine [51]. Future studies in larger and more heterogenous patient populations need to confirm this preliminary observation. The characteristic lag time in classical antidepressant effectiveness has given impulse to the search for novel therapeutics with more rapid effects. In such respect, the finding of ketamine as a rapid antidepressant in drug resistant patients has been a major breakthrough [99,100,101]. Unfortunately, the drug is characterized by adverse effects that limit its use [102]. Unlike ketamine, LAC has a high tolerability profile, and it is considered safe in humans [62]. In principle, this could allow its employment even in patient subpopulations who are very sensitive to the side effects associated with classical monoaminergic antidepressant drugs. LAC clinical studies were mainly performed in elderly patients. A recent meta-analysis confirmed that LAC is indeed more effective in older than in younger patients [61]. At present, no clear explanation is available for such a peculiarity of LAC. Although research efforts are required to confirm these findings, they suggest that LAC may represent a potential alternative to classical antidepressants. In particular, the elderly population and patients with comorbid medical conditions that make them vulnerable to adverse drug effects could represent an ideal subpopulation for LAC administration. In the future, it may also be interesting to assess whether LAC may produce antidepressant effects in subpopulations of drug-resistant patients.

**Figure 2:**
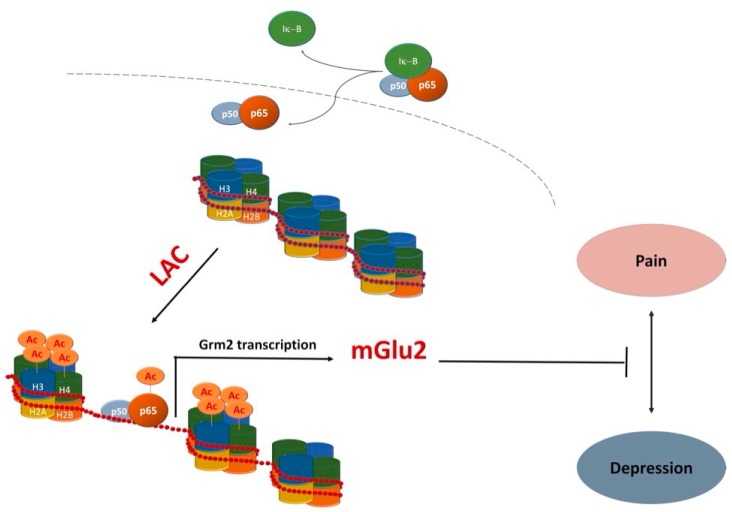
One potentially common mechanism underlying antidepressant and analgesic effects of LAC. LAC enhances *mGlu2* receptor (Grm2) gene transcription by acting as a donor of acetyl groups to NF-κB p65 and histones H3 and H4 [7,8,51,53,56].

LAC is currently marketed for treatment of neuropathic pain. Several studies, including double-blind placebo-controlled studies, have shown that LAC may represent a consistent therapeutic option for peripheral neuropathies [52,103]. As previously mentioned, LAC-mediated modulation of mGlu2 gene expression via NF-κB p65 acetylation has been proposed as a major contributor to its analgesic effects [7,50,56,103]. Recently, it has been shown that LAC, compared to other effective analgesic drugs, can also result in a very long-lasting analgesic effect in experimental models of both chronic inflammatory and neuropathic pain [53]. Once again, LAC long lasting effects were associated with an increase in mGlu2 receptor protein levels in the dorsal horns of spinal cord [53]. These observations have important clinical implications in view of the possibility that the drug might also reduce relapses in patients suffering from chronic pain. In light of the frequent comorbidity between depression and chronic pain [104], LAC exerting antidepressant and analgesic effects via common mechanisms (Figure 2) may represent an ideal treatment option.

In summary, for a long time there have been few drugs mechanistically distinct from classical monoaminergic antidepressants for treatment of mood disorder. LAC represents a next-generation antidepressant drug with novel mechanisms of action and high tolerability. Future investigations of the detailed cellular and molecular mechanisms underlying LAC effects may help in the development of potentially rapidly acting therapeutics and, in parallel, increase our current knowledge on the pathophysiology of depressive disorders.

## Figures and Tables

**Table 1:** l-Acetylcarnitine (LAC) clinical studies for disorders of central and peripheral nervous system.

Clinical Condition	References
Alzheimer’s disease	[9,10,11,12]
Parkinson’s disease	[13]
Huntington’s disease	[14]
Down’s syndrome	[15]
Dysthymic/Depressive disorder	[16,17]
Diabetic neuropathy	[18,19,20,21]
HIV Neuropathy	[22]
Carpal tunnel syndrome	[23]
Fibromyalgia	[24,25]

## Abbreviations

ahNG	Adult Hippocampal Neurogenesis
AcetylCoA	Acetyl-Coenzyme A (acetyl-CoA)
ahNPC	Adult Hippocampal Neural Progenitor Cell
BDNF	Brain Derived Growth Factor
CACT	Carnitine/Acetylcarnitine Translocase
CAT	Carnitine Acetyltransferase
ChAT	Choline Acetyltransferase
FSL	Flinders Sensitive Line
FRL	Flinders Resistant Line
GABA	Gamma-aminobutyric Acid
HAT	Histone Acetyltransferase
HDAC	Histone Deacetylase
Lys	Lysine
i.c.v.	Intracerebroventricular
LAC	l-AcetylCarnitine
Lepr	Leptin receptor
MDD	Major Depressive Disorder
mGlu2	Metabotropic Glutamate Receptor type-2
NAc	Nucleus Accumbens
MR	Mineralcorticoid Receptor
NPC	Neural Progenitor Cell
NF-κB	Nuclear Factor in the Kappa Light Chain Enhancer of B cells
OCTN2	Organic Cation/Carnitine Transporter
RCT	Randomized Clinical Trials
UCMS	Unpredictable Chronic Mild Stress
